# Enhanced Panoramic Radiograph-Based Tooth Segmentation and Identification Using an Attention Gate-Based Encoder–Decoder Network

**DOI:** 10.3390/diagnostics14232719

**Published:** 2024-12-03

**Authors:** Salih Taha Alperen Özçelik, Hüseyin Üzen, Abdulkadir Şengür, Hüseyin Fırat, Muammer Türkoğlu, Adalet Çelebi, Sema Gül, Nebras M. Sobahi

**Affiliations:** 1 Department of Electrical and Electronics Engineering, Faculty of Engineering, Bingöl University, Bingöl 12000, Turkey; sozcelik@bingol.edu.tr; 2 Department of Computer Engineering, Faculty of Engineering, Bingöl University, Bingöl 12000, Turkey; huzen@bingol.edu.tr; 3 Department of Electrical and Electronic Engineering, Faculty of Technology, Firat University, Elazig 23000, Turkey; 4 Department of Computer Engineering, Faculty of Engineering, Dicle University, Diyarbakir 21000, Turkey; huseyin.firat@dicle.edu.tr; 5 Department of Software Engineering, Samsun University, Samsun 55000, Turkey; muammer.turkoglu@samsun.edu.tr; 6 Oral and Maxillofacial Surgery Department, Faculty of Dentistry, Mersin University, Mersin 33000, Turkey; adalet_celebi@hotmail.com; 7 Department of Physiotherapy and Rehabilitation, Faculty of Health Sciences, Ondokuz Mayis University, Samsun 55000, Turkey; sema.gul@omu.edu.tr; 8 Department of Electrical and Electronics Engineering, Faculty of Engineering, King Abdulaziz University, Jeddah 21589, Saudi Arabia; nsobahi@kau.edu.sa

**Keywords:** tooth segmentation, tooth labelling, squeeze and excitation, attention gate, encoder–decoder

## Abstract

Background: Dental disorders are one of the most important health problems, affecting billions of people all over the world. Early diagnosis is important for effective treatment planning. Precise dental disease segmentation requires reliable tooth numbering, which may be prone to errors if performed manually. These steps can be automated using artificial intelligence, which may provide fast and accurate results. Among the AI methodologies, deep learning has recently shown excellent performance in dental image processing, allowing effective tooth segmentation and numbering. Methods: This paper proposes the Squeeze and Excitation Inception Block-based Encoder–Decoder (SE-IB-ED) network for teeth segmentation in panoramic X-ray images. It combines the InceptionV3 model for encoding with a custom decoder for feature integration and segmentation, using pointwise convolution and an attention mechanism. A dataset of 313 panoramic radiographs from private clinics was annotated using the Fédération Dentaire Internationale (FDI) system. PSPL and SAM augmented the annotation precision and effectiveness, with SAM automating teeth labeling and subsequently applying manual corrections. Results: The proposed SE-IB-ED network was trained and tested using 80% training and 20% testing of the dataset, respectively. Data augmentation techniques were employed during training. It outperformed the state-of-the-art models with a very high F1-score of 92.65%, mIoU of 86.38%, and 92.84% in terms of accuracy, precision of 92.49%, and recall of 99.92% in the segmentation of teeth. Conclusions: According to the results obtained, the proposed method has great potential for the accurate segmentation of all teeth regions and backgrounds in panoramic X-ray images.

## 1. Introduction

Dental diseases are a serious health problem that can cause deterioration in oral and dental health, speech disorders, and aesthetic problems [1]. According to the World Health Organization, oral diseases such as tooth decay, gum problems, oral cancer, and tooth loss affect approximately 3.5 billion people, nearly half the world’s population [2]. For this reason, early diagnosis of dental diseases is vital and can be assistive in effective treatment planning [3]. 

Tooth numbering performed with high precision is a fundamental requirement for the accurate segmentation of dental diseases and effective treatment planning [4]. This process is usually performed manually by a specialist dentist. The relevant specialist dentist carefully examines the patient’s mouth and dental structure and detects missing teeth. Although these procedures have been made easier using imaging methods such as panoramic radiography, they still rely on interpreting the human eye. Therefore, expert dentists may make mistakes when interpreting dental radiographs [5,6]. These errors can lead to negative consequences, including delayed disease diagnosis, unnecessary treatment, adverse outcomes, and even patient death [7,8,9]. 

It is stated that artificial intelligence (AI) will play an important role in automating routine tasks and increasing efficiency in healthcare [10]. AI is achieving important results in dentistry, as in other health fields. In particular, deep learning methods can perform complex operations such as teeth segmentation. This approach, which can provide accurate and rapid results regarding dental health by analyzing dental images in detail, is said to have great potential [11,12,13]. This tool makes it possible to develop auxiliary support systems for dental professionals. Based on these systems, dentists may perform tooth numbering and missing tooth detection in a shorter time and with high accuracy. Precise segmentation and numbering of teeth using deep learning can both reduce the error rate of specialist dentists and help plan treatment more quickly and effectively. 

The development of deep learning-based methods in dentistry and dental scanning technologies has gained significant momentum in recent years. Park et al. [14] employed segmentation and detection models to successfully identify missing tooth regions in panoramic radiographic images, achieving average precision values between 92.14% and 59.09%. Similarly, Chen et al. [11] proposed a TensorFlow-based Faster R-Convolutional Neural Network (Faster R-CNN) model for detecting and numbering teeth in dental periapical films, attaining over 90% accuracy in both precision and recall rates. Estai et al. [12] developed a three-step method based on CNN for 591 digital orthopantomography (OPG) images collected from patients over 18 years, achieving high recall and precision rates of 99% for tooth detection and numbering. Im et al. [15] introduced a dynamic graph convolutional neural network (DGCNN)-based algorithm for automatic tooth segmentation and classification in digital tooth models, reaching a segmentation success rate of 97.26%. Kilic et al. [16] presented an artificial intelligence system for detecting and numbering milk teeth, demonstrating impressive results with 98.04% precision and 96.86% F1 score. Krois et al. [17] observed a significant increase in classification accuracy in panoramic dental radiographs as contextual information increased. Karaoglu et al. [18] used a Mask R-CNN and heuristic algorithm-based method to evaluate tooth numbering in panoramic dental radiography images, achieving a mean Average Precision (mAP) of 92.49%. Other studies have demonstrated the effectiveness of deep learning-based techniques in various subfields of dentistry. Hao et al. [19] achieved 96.94% accuracy with a model trained on intraoral scanned data. Wu et al. [20] proposed a mesh-based deep learning framework for tooth labeling and reference point identification in raw intraoral scans. This framework achieved successful results with a 0.964 Dice similarity coefficient in segmentation, achieving a mean absolute error of 0.597 mm in reference point identification. Silva et al. [21] conducted a study focusing on tooth segmentation, numbering, and sample segmentation using the UFBA-UESC Tooth Images Deep dataset, finding that the PANet architecture provided the best results with 71.3% in segmentation and 74.0% mAP in numbering. Chandrashekar et al. [22] developed a collaborative deep learning model for tooth segmentation and identification using the UFBA-UESC tooth dataset. The proposed collaborative deep learning model was significantly more effective than individual models for tooth segmentation (98.77% vs. 96%) and identification (98.44% vs. 91%). Using a two-stage region proposal network, Tekin et al. [23] developed a segmentation network for bitewing dental radiographs. They worked with 1200 real-world data and achieved effective accuracy with specific hyper-parameter settings. The results with Mask R-CNN showed 100% accuracy and 97.49% mAP for tooth detection, 94.35% accuracy, and 91.51% mAP for tooth numbering. Current studies in the literature demonstrate the accuracy and effectiveness of artificial intelligence in detecting dental caries, dental calculus segmentation, and diagnosing general dental diseases in radiographs. Through the use of models such as U-Net, MAPPNet, and other hybrid approaches, these studies have achieved enhanced identification of dental anomalies, improved segmentation precision, and accelerated medical imaging processes. These models, by achieving higher accuracy in tooth segmentation, have emerged as supportive tools in clinical applications [24,25,26,27,28,29]. Unlike segmentation architectures, object detection models like YOLO are limited in capturing edge details at a pixel level, which are critical for the exact delineation of tooth boundaries. Segmentation models capture fine edge features well and represent them at a pixel level, allowing accurate tooth segmentation, especially in cases where teeth are overlapped. This is another important difference that makes the segmentation models more appropriate for tasks like ours, which require precision in defining the exact boundaries of dental structures.

Furthermore, while object detection tasks often face speed constraints, this is not of primary concern in the study at hand. The proposed SE-IB-ED model offers a satisfactory computational efficiency, as illustrated by its processing speed: 27.67 FPS on panoramic radiographs. However, this paper examines static, two-dimensional panoramic radiographs rather than video-like data, and it is more important to attain high accuracy in segmentation tasks than to perform with detection speed. Thus, this SE-IB-ED architecture is chosen with a special design for precise segmentation requirements and without aiming for the highest possible speeds. Table 1 provides a summary of recent studies focusing on teeth segmentation and numbering in dental radiographs. These studies highlight the advancements in artificial intelligence and deep learning techniques for improving segmentation precision and efficiency.

These studies demonstrate the effectiveness of deep learning-based methods in dentistry and digital dental scanning and their potential applications in these fields. The methods developed for automatic tooth detection, segmentation, and numbering can potentially increase efficiency in dental practices and provide more accurate diagnoses. Although good results have been achieved, most segmentation studies have been labeled using Bounding Box (BB). Unlike many others in the literature in this study, we utilized polygonal semantic pixel labeling (PSPL) for pixel-level annotation. PSPL offers more precise tooth boundary representation and cleaner data with reduced background noise compared to the BB method, and it is particularly effective in identifying overlapping teeth. In this study, a novel SE-IB-ED model has been developed, incorporating advanced feature extraction and segmentation techniques. This model aims to overcome the limitations of traditional methods by providing precise tooth boundary detection and improved performance in segmenting overlapping teeth. 

The main contributions of this study are as follows:

1. The SE-IB-ED model’s encoder–decoder structure is crucial to the process of extracting features and turning them into segmentation. In comparison to other new technology networks (Eff-U-Net, Att-U-Net, LinkNet, FPN, Trans-U-Net), this offers a more accurate segmentation.
2. The decoder of the model was able to concentrate and identify tooth boundaries more accurately due to the SE-based inception block technique.
3. 313 panoramic radiography images were obtained from private dental practices.
4. A more accurate model to segment the overlapping teeth has been constructed with the application of the PSPL and sigmoid activation function.

In the continuation of the study, Section 2 provides detailed information on the dataset created, the labeling method, and the developed network architecture. Section 3 presents the results obtained for each tooth using the proposed Squeeze and Excitation Inception Block-based Encoder–Decoder (SE-IB-ED) network architecture and compares the outcomes of the sigmoid and SoftMax activation functions, offering interpretations of these comparisons. Section 4 compares the SE-IB-ED model with the latest technology in segmentation methods and discusses the results. In Section 5, the conclusion is presented.

## 2. Proposed Methodology

A total of 500 panoramic radiography images were obtained from private dental clinics. Out of these, 313 images were selected after removing patients with conditions such as cysts and jaw fractures and those who were outside the age range of 18 to 65 years. Employing the PSPL labeling method, we annotated 32 teeth according to the FDI [30] numbering system, treating each tooth as a distinct class. In addition, a novel methodology for segmentation of the teeth from panoramic dental X-ray images is proposed.

**Figure 1:**
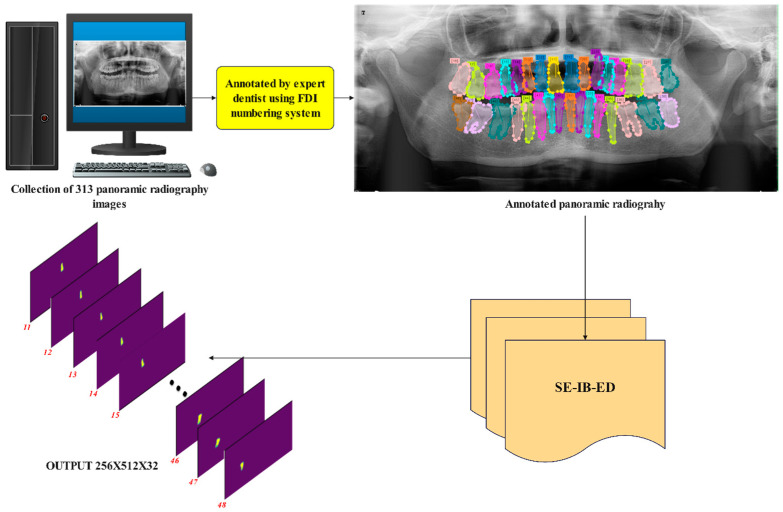
Proposed system.

The system applied in this study is presented in Figure 1. The proposed system essentially consists of three main stages. First, panoramic dental images were collected, and annotations were added to the teeth. Then, a new encoder–decoder architecture was developed for detailed tooth segmentation. In the encoder section of the developed model, the Inception V3 model was used for powerful initial featuring. Feature maps are retrieved from the five intermediate layers of the Inception V3 model and sent to the decoder. Pointwise convolution and upsampling are used inside the decoder to combine the information. An attention-gating mechanism based on Squeeze and Excitation Inception Block (SE-IB) is then used to improve feature integration. After feature fusion, the final feature map is created by applying several convolution layers. In the last part of the proposed framework, a pixel-level convolution layer and sigmoid function are used to generate a 32-channel tooth segmentation output. Many experimental studies have been conducted using the proposed model. In experimental studies, it has been observed that the proposed model exhibits superior performance for tooth segmentation.

**Figure 2:**
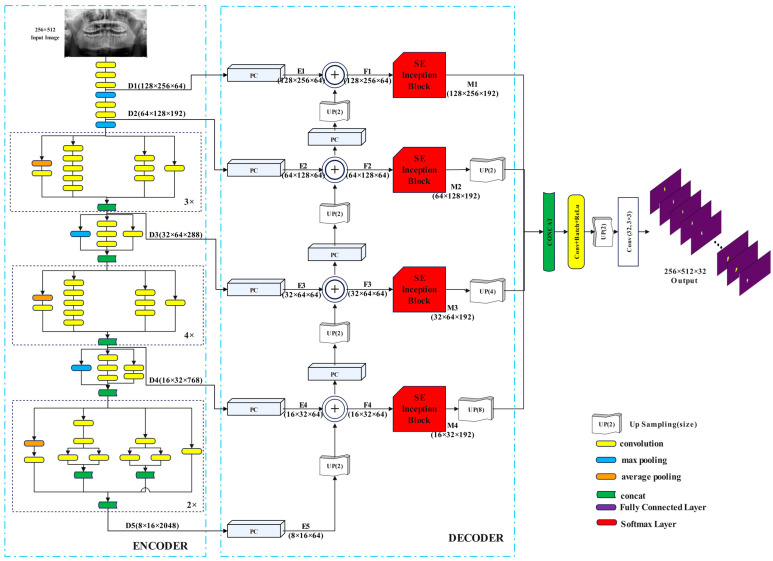
SE-IB-ED architecture.

This study introduces a novel network architecture designed for segmenting teeth from panoramic dental X-ray images. This architecture incorporates an encoder for extracting robust initial features and a decoder for efficiently processing them. The SE-IB-ED model is illustrated in Figure 2.

Figure 2 shows that the encoder component of the proposed model utilizes the InceptionV3 architecture, from which five levels of features (D_1_–D_5_), both low and high, are extracted. These feature maps are then passed to the decoder, where pointwise convolution is applied to standardize the depth dimensions (E_1_–E_5_). Subsequently, up-sampling and element-wise addition are employed for feature integration. This process is hierarchically repeated to obtain four feature maps (F_1_–F_4_). In the second part of the decoder, a squeeze and excitation-based inception block is applied to the F_1_–F_4_ features. The output of these blocks yields M_1_–M_4_ feature maps in the decoder part. Finally, all decoder features are resized to uniform dimensions via upsampling and combined to generate the final feature map. The final stage of the proposed model employs a sigmoid-based multi-channel segmentation module. After applying a series of convolution and upsampling layers to the final feature map, this module produces an output with dimensions of 256 × 512 × 32, where each channel represents a tooth. At the end of the module, the sigmoid function is applied to the output to achieve independent scores for overlapping teeth. As a result, the network architecture produces segmentation predictions with independent outputs for each tooth.

### 2.1. Encoder

**Figure 3:**
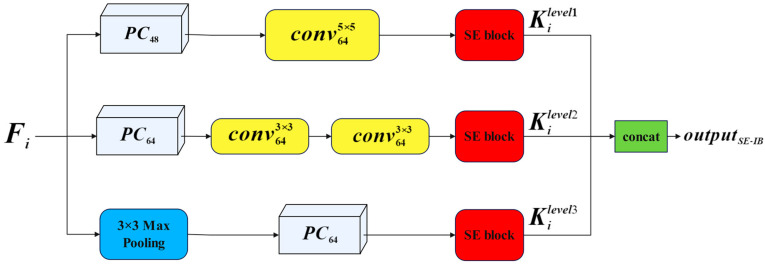
Squeeze-and-excitation-based Inception block (SE-IB).

The proposed architecture’s encoder component uses a multi-level feature extraction design. Obtaining low-level and high-level features from the input image is the main goal of this architecture. Low-level features include details like edge color and texture, but high-level features contain semantic information, as stated by Ronneberger et al. [31]. Similarly, tooth segmentation semantic information helps with the exact segmentation of the tooth region, while edge, color, and texture details help with correct tooth region localization. In light of this, the suggested model uses the pre-trained InceptionV3 network architecture to extract potent low- and high-level features. The primary justification for utilizing InceptionV3 as the encoder is its inception blocks [32,33,34], which make it possible to extract features of different sizes and produce a rich feature map. The InceptionV3 network architecture is detailed in Figure 3, where convolution operations are shown in yellow, maximum pooling in blue, and average pooling in orange. Five layers (D_1_–D_5_) of features are extracted from the InceptionV3 architecture using a 256 × 512 panoramic X-ray picture as input. Regions with lower feature dimensions are usually used for feature selection. This is because it makes it possible to extract features at various resolutions, producing feature maps that are both semantically and geographically rich. Figure 3 provides comprehensive details on the D_1_, D_2_, D_3_, D_4_, and D_5_ feature map from the encoder part to the decoder. The corresponding dimensions of these features are 128 × 256 × 64 (D_1_), 64 × 128 × 192 (D_2_), 32 × 64 × 288 (D_3_), 16 × 32 × 768 (D_4_), and 8 × 16 × 2048 (D_5_).

### 2.2. Decoder

The Decoder block consists of three main sections: feature integration, the attention gate part of the SE Inception block, and multi-channel segmentation output.

#### 2.2.1. Feature Integration

The feature maps *D*_1_–*D*_5_ transferred to the decoder component have different resolutions and depths. Inspired by the Feature pyramid network (FPN) [35] model to associate these attributes, a Feature Integration Block (FIB) is utilized. In the FIB, pointwise convolution [36] is initially applied to low- and high-level features, thereby equalizing the depths of the features.

(1) $$E_{i}=PC^{128}\left(D_{i}\right)\;i\in \left\{1,2,3,4,5\right\}$$

Here, the provided *PC* denotes the pointwise convolution layer. The number of filters in the PC is 128. As a result of this operation, feature maps *E*_1_, *E*_2_, *E*_3_, *E*_4_, and *E*_5_, all with the same depth dimension, are obtained. Subsequently, these features are hierarchically associated according to the following equation.

(2) $$F_{i-1}=Up^{2}\left(F_{i}\right)⨁E_{i-1}\;i\in \left\{2,3,4,5\right\}$$

The symbols ⨁ and *Up* indicate element-wise addition and upsampling layers, respectively. The size of the upsampling kernel is 2. The high-level feature map at the *i_th_* level, represented by the supplied $F_{i}$, will have values of 5, 4, 3, 2, and so on. The F5 feature map’s value is also initialized to *E*_5_ (*F*_5_ = *E*_5_). After that, the upsampling layer is used to resize the $F_{i}$ feature map for $F_{i-1}$ to the same size as $E_{i-1}$ and *E_i_* is added elementwise. The main objective is to transmit the information from high-level characteristics to low-level features hierarchically, akin to the U-Net paradigm. In contrast to U-Net, it is less computationally expensive to use elementwise addition rather than concatenation. As a result of this process, feature maps *F*_1_, *F*_2_, *F*_3_, *F*_4_ are obtained.

#### 2.2.2. Squeeze-and-Excitation-Based Inception Block

An attention gate based on the squeezing and excitation process [37], termed SE-IB, operates on the feature maps *F*_1_–*F*_4_. SE-IB is depicted in red in Figure 2 and is further illustrated in detail in Figure 3.

The mathematical expression of the SE-IB block given in Figure 3 is given in Equation (3):

(3) $$K_{i}^{level1}=Conv^{5\times 5}\left(PC\left(F_{i}\right)\right)\;K_{i}^{level2}=Conv^{3\times 3}\left(Conv^{3\times 3}\left(PC\left(F_{i}\right)\right)\right)\;K_{i}^{level3}=PC\left(MaxPooling\left(F_{i}\right)\right)\;output_{\mathrm{SE}-\mathrm{IB}}=concat\left[\begin{array}{c} \;SE\left(K_{i}^{level1}\right)\\ SE\left(K_{i}^{level2}\right)\\ SE\left(K_{i}^{level3}\right) \end{array}\right]$$

The provided F_i_ represents the feature map transferred to the Inception block. The proposed Inception block consists of 3 levels, and their outputs are denoted as $K_{i}^{level1}$, $K_{i}^{level2}$, and $K_{i}^{level3}$. Here, various convolution operations with different kernel sizes are applied for each level. Subsequently, the proposed SE attention gate is applied for each level. Finally, the obtained features are merged in the depth dimension. The formulation of the SE block structure used in this study is provided in Equation (4):

(4) $$cf=PC^{c\;}\left(PC^{\frac{c}{2}\;}\left(K\right)\right)\;scf=sigmoid\left(cf\right)\;WK=scf⨂K$$

Here, the given *PC* represents pointwise convolution. *c*/2 and *c* denote the number of filters applied for squeeze and expansion, respectively, with c = 64. Additionally, cf and scf represent the weight coefficient and scaled weight coefficient, respectively. Two consecutive PC operations are applied to the input feature map *K* transferred to the SE block shown in Equation (4). The resulting PC outputs are utilized as feature weights. These obtained values are then subjected to a sigmoid activation function to be weighted between 0 and 1. Consequently, scaled coefficients (*scf*) varying between 0 and 1 are obtained. Finally, elementwise multiplication (⨂) is performed between scf and the *K* feature. As a result, the input *D*_1_*–D*_5_ features to the decoder section are transformed into *F*_1_*–F*_4_ features through a series of convolution and elementwise addition operations. Subsequently, each *F* feature is passed through the SE-IB attention gate to obtain *M*_1_–*M*_4_ features. The resulting *M*_1_*–M*_4_ features are then transferred to the multi-channel segmentation output.

#### 2.2.3. Multi-Channel Segmentation Output

The *M*_1_–*M*_4_ outputs from the proposed SE-IB were used for segmentation output prediction. These features are of sizes 128 × 256 × 192, 64 × 128 × 192, 32 × 64 × 192, and 16 × 32 × 192, respectively. To concatenate the *M*_1_–*M*_4_ features, the first and second dimensions need to be resized to the same size. Therefore, an upsampling layer was used to bring the smaller dimensions to the largest size of 128 × 256. A concatenation layer was employed for the merging process, as shown in Equation (5):

(5) $$F_{final}=concat\left[\begin{array}{c} \begin{array}{c} \;M_{1}^{128\times 256\times 192}\\ UP^{2}\left(M_{2}^{64\times 128\times 192}\right)\\ UP^{4}\left(M_{3}^{32\times 64\times 192}\right) \end{array}\\ UP^{8}\left(M_{4}^{16\times 32\times 192}\right) \end{array}\right]$$

In the proposed model, the final operation results in obtaining a multichannel segmentation output using the sigmoid function. This process is presented in Equation (6).

(6) $$Prediction\;output=Sigmoid\left(Conv^{32}\left(Up^{2}\left(ConvbatchRelu\left(F_{final}\right)\right)\right)\right)$$

As shown here, the *F_final_* feature map is first subjected to *Conv*, *Batch*, and *ReLu* layers (*ConvBatchRelu*(*F_final_*)). Then, to match the output size to the image size (256 × 512), an upsampling with a kernel size of 2 is applied. Finally, to obtain the segmentation output, a convolution layer and sigmoid activation function are applied. The number of filters for this convolutional layer (*Conv*^32^) is 32 (the number of teeth).

The main purpose of using sigmoid activation in the proposed model is to generate independent outputs for each channel. This is particularly important for overlapping teeth, as SoftMax activation calculates a probabilistic distribution between 0 and 1 based on values in all channels. However, the same pixel is correct for overlapping teeth in two different channels, leading to incorrect results with SoftMax. In contrast, the sigmoid function directly compresses the prediction output between 0 and 1, allowing each channel to act independently.

Both SoftMax and sigmoid activation functions have been used for analysis in experimental studies. Results have shown that the sigmoid activation function is more effective. The expression of the sigmoid and SoftMax activation function is given in Equation (7).

(7) $$sigmoid\left(x\right)=\frac{1}{1+e^{-x}}\;softmax\left(x\right)=\frac{e^{x}}{\sum_{j=1}^{n}e^{x_{j}}}$$

## 3. Experimental Works

Experimental studies were conducted on an RTX 3080Ti, i9 processor, and 64 GB RAM. A total of 313 labeled panoramic dental radiograph images were resized to 256 × 512 dimensions. The data were split into 80% for training (251 images) and 20% for testing (62 images). Data augmentation was applied. The dataset was trained and tested on our own developed SE-IB-ED, as detailed in Section 4. Additionally, it was trained and tested against models like Eff-Unet [38], Attention Unet [39], LinkNet [40], Feature Pyramid Network [35], Trans Unet [41], Unet [32], and Swin-Unet [42] for comparison.

### 3.1. Dataset and Ground Truth Annotations

This retrospective study was approved by the Ethics Committee (2023/13-37). A total of 500 panoramic radiography images were anonymously collected to ensure the confidentiality of patient personal information, such as gender, name, age, etc. A total of 313 images were selected, excluding patients between 18 and 65 and patients with conditions such as cysts and jaw fractures. Each panoramic radiograph was saved as an image file in ‘jpeg’ format with a resolution of approximately (2700 to 3000) × (1316) pixels with a depth of 8 bits. An oral and maxillofacial surgeon labeled all the teeth in the obtained images with over 10 years of experience. The labeling process utilized the FDI tooth numbering system. The tooth numbering system developed by the Fédération Dentaire Internationale (FDI) is a globally accepted identification methodology in the field of odontology. This system divides the oral cavity into four anatomical quadrants: upper right, upper left, lower right, and lower left. A unique numeric value represents each quadrant: 1 for the upper right, 2 for the upper left, 3 for the lower left, and 4 for the right. The dentition in these quadrants is sequentially numbered, starting from the first dental to the molaris dentium. The system assigns a two-digit numeric code to each tooth: the first digit indicates the quadrant, and the second digit indicates the tooth’s position within that quadrant. For instance, the second molar in the upper right jaw is coded as ‘17’ (first quadrant, seventh tooth), while the first incisor in the upper left jaw is coded as ‘21’ (second quadrant, first tooth). A table of the FDI tooth numbering system is provided in Table 2.

BB is a method where the tooth is labeled by enclosing it within a rectangular box. Polygonal Semantic Pixel Labeling (PSPL), which involves labeling around all lines of the tooth (crown and root), has been less favored by researchers as it is time-consuming compared to BB. The advantages of PSPL over BB are listed:

1. PSPL follows the true boundaries of the tooth more precisely, while rectangular BB does not fully conform to the tooth’s outer lines.
2. In the BB method since the tooth is enclosed in a rectangle, it includes unnecessary background information, whereas the PSPL method provides cleaner data by minimizing unnecessary background information.
3. PSPL distinctly identifies overlapping teeth separately, while BB may struggle in separation.

**Figure 4:**
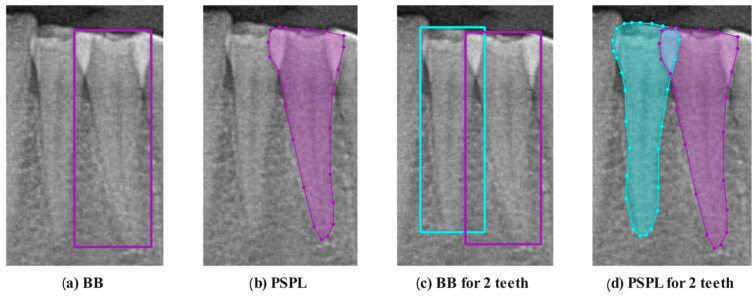
(**a**) A tooth image labeled with a BB. (**b**) A tooth image labeled with a PSPL. (**c**) An image of two overlapping teeth labeled with BB. (**d**) An image of two overlapping teeth labeled with PSPL.

In Figure 4, the same tooth is labeled by an expert dentist using both BB (a) and PSPL (b). When examining Figure 4c, it is clearer to understand most of the disadvantages mentioned above, such as unnecessary background information and overlapping teeth, when two adjacent teeth are labeled using the BB method. In Figure 4d, however, when two adjacent teeth are labeled with PSPL, a smoother labeling is presented that can overcome the problems of unnecessary background and overlapping of teeth.

**Figure 5:**
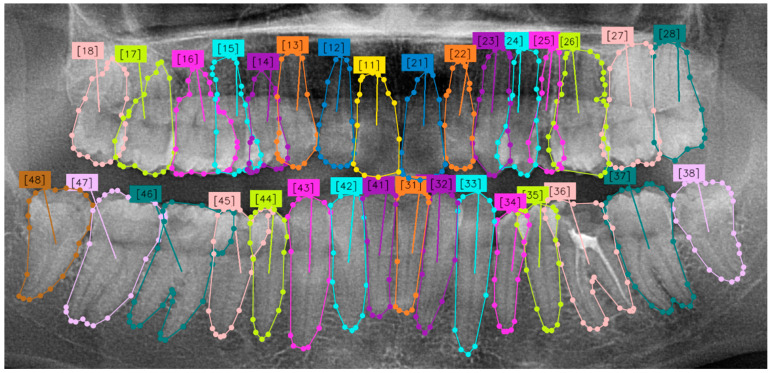
A panoramic radiograph annotated by the expert dentist according to the FDI numbering system.

In this study, instead of manual labeling with PSPL, the Segment Everything Model (SAM) algorithm [43], which can be performed faster and more smoothly, was used. The SAM accelerates the process significantly through its automatic labeling feature in problems requiring numerous different labels in an image. However, it creates errors in the root and crown parts of the teeth. Erroneous sections were corrected manually. As a result, a pixel-level labeled mask was obtained for each panoramic dental radiograph. Figure 5 presents an example tooth image labeled according to the FDI system by our expert dentist. 

### 3.2. Performance Metrics

The efficiency of the proposed model is determined using performance metrics such as precision, F1-score, recall, and Intersection over Union (IoU) [44]. The F1 score is a particularly suitable metric for assessing success in segmentation because it balances the model’s false positives and false negatives. Here, false positives represent pixels that are incorrectly classified, while false negatives are pixels that are erroneously missed. The F1 score is calculated for an object class or region in segmentation. This involves pixel-based evaluation of true positives, false positives, and false negatives. To calculate the F1 score, precision and recall values are needed [45]. 

Precision: It is the ratio of the pixels correctly identified by the model as belonging to the target class to the total number of pixels predicted as belonging to that class during segmentation. The calculation of precision is given in Equation (8) [46].

(8) $$\mathrm{Precision}=\frac{\mathrm{TP}}{\mathrm{TP}+\mathrm{FP}}$$

Recall: It is the ratio of the pixels that the model correctly identifies as belonging to the target class to the total number of pixels that actually belong to that class. The calculation of recall is given in Equation (9) [46].

(9) $$\mathrm{Recall}=\frac{\mathrm{TP}}{\mathrm{TP}+\mathrm{FN}}$$

True Positive (TP): The pixels the model correctly identifies as belonging to the target class.

False Positive (FP): The pixels the model incorrectly marks as belonging to the target class but, in reality, are not.

False Negative (FN): The pixels that belong to the target class but are missed or misclassified by the model.

F1 Score: It is the harmonic mean of precision and recall. The calculation of the F1 score is given in Equation (10).

(10) $$F1-\mathrm{score}=2\;\times \;\frac{\mathrm{Precision}\;\times \;\mathrm{Recall}}{\mathrm{Precision}+\mathrm{Recall}}$$

Intersection over Union (IoU) is a metric that determines how much the segmentation area predicted by the model overlaps with the actual correct segmentation area. Fundamentally, it can be defined as the ratio of the intersection area between the predicted region and the actual region to the union area of these two regions. The calculation of IoU is given in Equation (11).

(11) $$\mathrm{IoU}=\frac{\mathrm{Object}\;\cap \;\mathrm{Detected}\;\mathrm{Area}}{\mathrm{Object}\;\cup \;\mathrm{Detected}\;\mathrm{Area}}$$

Intersection Area: This is the overlapping part of the segmentation area determined by the model and the actual segmentation area. 

Union Area: This refers to the total area encompassed by both the segmentation area predicted by the model and the actual segmentation area combined.

### 3.3. Results

The proposed SE-IB-ED architecture has the capacity to recognize 32 different tooth classes. The performance of the model has been evaluated using five main metrics: F1-score, mean Intersection over Union (mIoU), Precision, Recall, and Accuracy. These metrics provide a comprehensive assessment of the model’s segmentation capability. The results obtained demonstrate the success of the proposed architecture in addressing the tooth segmentation problem. The results are summarized in Table 3.

On average, an F1-score of 92.66% indicates that the model demonstrates a balanced performance in precision and recall. It is observed that F1-score values vary significantly between 88% and 94% according to tooth numbers, which shows the model’s capability to segment most tooth types with high accuracy. An average mIoU value of 86.39% illustrates how well the model’s segmentation aligns with the actual boundaries, indicating that the model generally captures the shape and boundaries of the teeth well. An average precision of 92.84% and an average recall of 92.49% suggest that the model’s segmentations are accurate (few false positives) and comprehensive (few false negatives). A balanced ratio between these two metrics demonstrates the consistency of the model’s performance. An average accuracy of 99.93% indicates that the model achieves high success in the segmentation task overall, demonstrating that it correctly classifies nearly all pixels. Some tooth numbers (11, 21, 33, 43, 44) stand out with particularly high F1-score and mIoU values, indicating better performance on specific tooth types. On the other hand, a lower performance has been observed for some teeth (for example, 14, 24, 25), which may be due to the more complex nature of segmenting these teeth.

In this study, we addressed the issue of overlapping teeth by using the sigmoid activation function in the network’s final layer for classification. The sigmoid function maps the predicted scores between 0 and 1. We then performed thresholding by applying a cutoff at t = 0.5, where values above 0.5 were designated as teeth, and all other values were set to 0. This thresholding was applied across all channels of the 256 × 512 × 32 output prediction matrix. As a result, each channel corresponds to a tooth, creating a prediction output. These prediction outputs were compared with the actual labels to calculate the F1 score, precision, recall, and accuracy for each tooth. Additionally, this table includes results obtained using the SoftMax activation function to evaluate its efficacy in dealing with overlapping teeth. For the SoftMax outcomes, the final layer of the proposed model was modified to incorporate the SoftMax function. The experimental setup was adjusted accordingly to accommodate the SoftMax function. The results of these comparisons are provided in Table 4.

**Figure 6:**
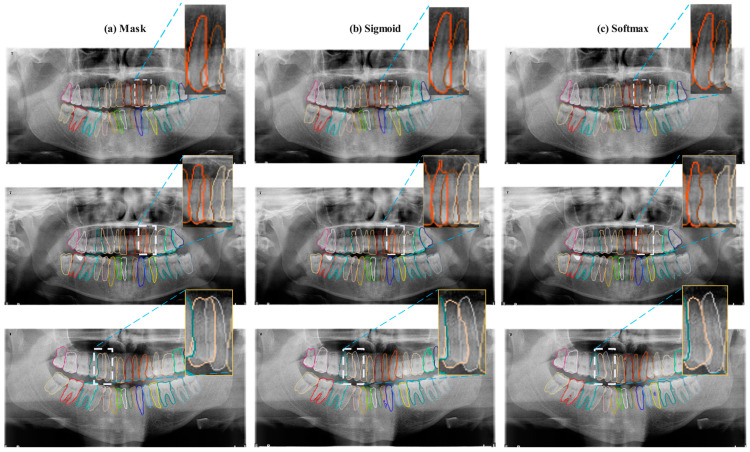
Comparative analysis of segmentation outcomes obtained for overlapping teeth using Sigmoid and SoftMax activation functions. Panel (**a**) displays the ground truth mask with clearly delineated tooth boundaries. Panels (**b**,**c**) illustrate the segmentation results using sigmoid and SoftMax activations, respectively.

As shown in Table 4, the sigmoid function has achieved higher success on average. The sigmoid function’s ability to individually assess each tooth has led to its higher success in overlapping tooth problems. In contrast, the SoftMax classifier, by forcing a pixel to belong to one tooth, must assign overlapping pixels to a single class, which can lead to incorrect identification of overlapping areas. This issue is evident in the results presented in Table 3. Specifically, for teeth with overlapping conditions such as teeth numbers 13–14, 27–28, and 36–37, the sigmoid activation function has achieved higher F1 scores. To further analyze these results, sample tooth images and the prediction results of the models are provided in Figure 6.

In Figure 6, comparative results of two different activation functions (sigmoid and SoftMax) used in segmenting overlapping teeth are presented. In the Mask (a) section, the ideal segmentation of each tooth is prominent, serving as the baseline reference point that represents the actual data against which the other two methods will be compared. The Sigmoid (b) and SoftMax (c) sections display the segmentation results obtained using these activation functions. In areas of tooth overlap, the segmentation by the sigmoid activation function appears to be clearer and more precise; this difference is particularly noticeable in regions marked with blue arrows. The sigmoid function, by treating each classification independently, has facilitated a more detailed definition of overlapping teeth.

The reasons why sigmoid may be stronger than SoftMax in segmenting overlapping teeth could include:

Since the sigmoid produces independent probabilities for each class, it allows for the modeling of multiple teeth’s presence in overlapping areas. For instance, if a pixel is located in an area where two different teeth overlap, the sigmoid can generate high values for both tooth classes. In SoftMax, however, the probabilities of all classes are interconnected, and as the probability of one class increases, the probabilities of the others decrease, making it harder to model overlapping areas.

The sigmoid function’s ability to generate independent values for each class provides more flexibility during training, allowing the model to learn class-specific details. This offers an advantage over SoftMax in overlapping classes.

In situations of class imbalance in the dataset (e.g., some teeth are more frequently observed than others), SoftMax could cause the model to favor dominant classes overly. Since Sigmoid considers each class independently, it might be more effective in learning about minority classes.

Table 5 provides a confusion matrix to evaluate the performance of the segmentation model. Each row represents an actual tooth number, while the columns represent the predictions made by the model. The first column, labeled “Tooth Number”, contains the actual tooth numbers, and the rest of the matrix contains the model’s pixel-based predictions.

At first glance, we can see that tooth number “11” has been identified with a high level of accuracy, with 36,448 correct classifications. For tooth number “12”, this number is 29,074. The tooth number “13” has also been correctly classified 35,299 times with high accuracy. However, the numbers outside the diagonal indicate that the model has confused some teeth with others. For instance, for tooth number “14”, 1647 pixels were mistakenly classified as belonging to tooth number “15”. This suggests a tendency for confusion between teeth numbers “14” and “15”. Generally, high numbers on the diagonal indicate the model’s ability to correctly recognize teeth, while high numbers outside the diagonal indicate false positives and potential confusion.

Table 6 compares the performances of various segmentation architectures with those of our proposed model, SE-IB-ED. In these comparisons, state-of-the-art models such as Eff-U-Net, Att-U-Net, LinkNet, FPN, and Trans-U-Net were used. In addition, all methods have been re-adjusted according to the proposed “multi-channel segmentation output” model for fair comparisons. In other words, the final layers of all models have been re-adjusted to provide high performance.

The SE-IB-ED model demonstrates superior performance among all models examined, achieving the highest values in five key performance metrics: F1-score, mIoU, Precision, Recall, and Accuracy. This indicates that the SE-IB-ED model offers a balanced performance in terms of both precision and sensitivity, capable of producing highly accurate results in segmentation tasks. While the Eff-U-Net and Att-U-Net models also show high performance, they rank behind SE-IB-ED. Their values in F1-score, mIoU, and other metrics suggest effectiveness in segmentation tasks, yet they do not reach the success level of SE-IB-ED. The LinkNet and FPN models display moderate performance, particularly falling short in F1-score and mIoU values. This suggests these models are less effective at determining segmentation boundaries as accurately as SE-IB-ED or Eff-U-Net. The Trans-U-Net model significantly underperforms in comparison to others, especially in the F1-score and mIoU metrics. This could imply that Trans-U-Net is less suitable for this specific segmentation task. 

**Figure 7:**
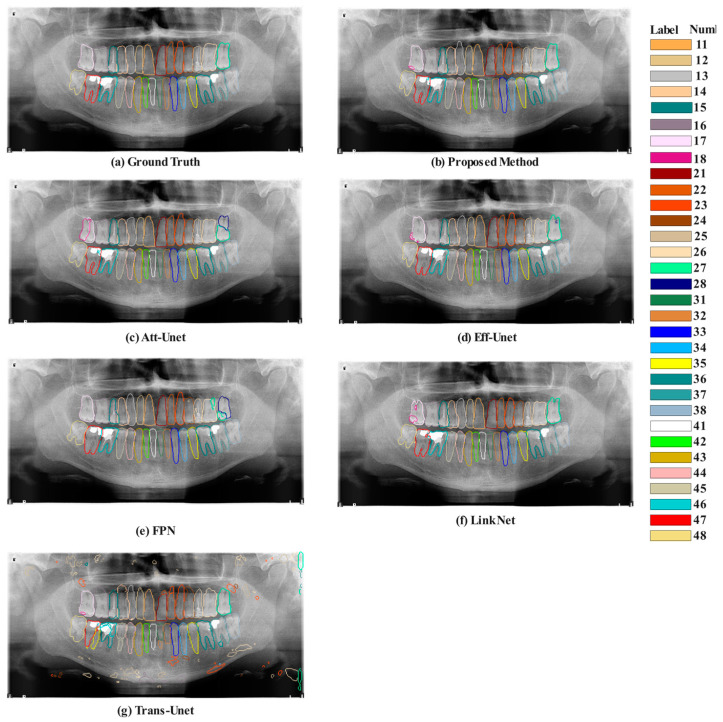
Comparative visualization of model performances obtained in the dental segmentation tasks.

Overall, this comparison highlights the SE-IB-ED model’s superiority in dental segmentation tasks over other pre-trained models, offering the most balanced performance. The image collection provided in Figure 7 visually demonstrates the performance of various models in the task of tooth segmentation.

Ground Truth serves as the benchmark for accuracy. It shows a detailed and precise demarcation of each tooth, providing a clear expectation for the segmentation models to aim for. The performance of the SE-IB-ED model appears to be nearly equivalent to the Ground Truth. Although it makes a minor error on teeth number 17, it seems to exhibit a very high level of accuracy. Att-U-Net shows decent accuracy, but there are some noticeable discrepancies. There seem to be uncertainties and misclassifications with teeth numbered 26 and 36. While Eff-U-Net generally seems to perform well, there are areas where the segmentation does not entirely align with the Ground Truth, particularly at the edges of some teeth, which may lead to less precise results. The performance of FPN varies across the image. In some areas, the segmentation is quite accurate, while in others, the delineation of teeth appears less defined, potentially leading to mixed accuracy in the results. Significant areas of miss segmentation are noticeable in LinkNet’s output. It seems to struggle with certain regions, potentially over-segmenting or missing some dental structures. Trans-U-Net shows a clear deviation from the expected results, with several teeth either not segmented at all or segmented with considerable inaccuracy, indicating a lower performance level.

## 4. Discussions

This paper proposes a new approach, SE-IB-ED, for segmenting teeth in panoramic X-ray images. The dataset consists of 313 panoramic radiography images collected from private dental clinics and annotated by the PSPL method with 32 teeth labeled based on the FDI numbering system. An innovative methodology for segmenting teeth in panoramic dental X-ray images was proposed. It consists of three main steps: (1) collection of images and annotation of teeth; (2) proposing a deep segmentation approach using an encoder–decoder architecture; and (3) refining the segmentation results by applying an attention gate module based on a SE-based Inception block. Experimental evaluation showed the effectiveness of the proposed method for the precise segmentation of 32 teeth in panoramic dental X-ray images. Table 7 summarizes the performance metrics of the SE-IB-ED model compared to other dental segmentation studies.

Table 7 shows the datasets used, the deep learning models applied, and the results obtained by various tooth segmentation studies. The proposed SE-IB-ED model is in a very good position in terms of accuracy metric compared to other models in the literature. Unlike the public datasets commonly used in the literature, a unique dataset containing challenging images was used. This dataset contains complex segmentation problems such as low-quality images, overlapping teeth, and missing teeth, which are frequently encountered in clinical applications. These results, obtained with a dataset of only 313 images, show that the model can work effectively in different scenarios. In particular, the new approach we developed for overlapping teeth problems is one of the unique contributions of our SE-IB-ED model to the field of tooth segmentation. Our model performed successfully even in cases where teeth overlap and segmentation becomes difficult. Although it lags behind some studies in terms of F1 score, our model performs well in difficult segmentation conditions. Furthermore, unlike most studies in the literature, we evaluated the segmentation accuracy of our model with the Intersection over Union (IoU) metric and demonstrated how well the segmentation mask and the real region overlap. These metrics again demonstrate the segmentation success of our model and its superior performance on complex oral structures. This indicates that the model can be a reliable tool for tooth segmentation in clinical applications.

The advantages of the study can be listed as follows:

- Although a large number of images were not used in the proposed model’s training phase, the model produced quite successful segmentations. This situation also reduces the related costs of data collection and labeling.
- The proposed model performs effective segmentations for the overlapping teeth based on the PSPL used in labeling the dataset and the sigmoid activation function used in the output of the proposed model.
- The balanced ratio between the obtained precision and recall metrics stands out. This indicates that the segmentation is achieved with the least number of false positives and false negatives.
- The disadvantages of the study are as follows:
- The proposed model produces false segmentations for the low-quality input images. To handle such a problem, a series of image enhancement operations can be used to improve the quality of such input images.
- Although the variety of data used in training is sufficient, 313 panoramic radiography images may be insufficient to represent other scenarios, such as different oral bone structures, cysts, and broken teeth. Expanding the dataset can handle this situation. It is already planned to expand this dataset and work on it in future studies.

**Figure 8:**
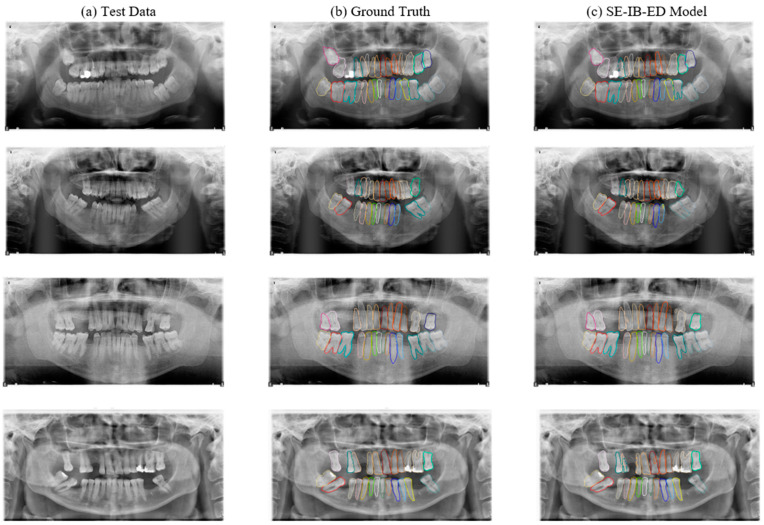
Performance evaluation of the SE-IB-ED model on challenging test images, including the original input, ground truth masks, and model predictions.

The SE-IB-ED model’s performance was evaluated under challenging conditions, such as images containing noise and artifacts. These conditions simulate real-world scenarios where image quality may not be ideal. Despite these challenges, the model demonstrated a robust segmentation capability, accurately delineating tooth boundaries in most cases. However, slight discrepancies were observed in certain regions with overlapping teeth or severe noise. Qualitative results showcasing the original input image, ground truth mask, and model predictions under such conditions are provided in Figure 8.

Figure 8 presents examples of segmentation results obtained using the SE-IB-ED model on challenging panoramic radiograph images and presents both realistic strengths and limitations. The input radiographs have several real-world artifacts and challenges, which include bright reflections and shadowing caused by metallic restorations, low contrast in some regions, overlapping teeth, and noise due to the imaging process. These can complicate the process of segmentation by obscuring the boundaries of the teeth or by presenting misleading features for the model.

In the second row, the model achieves effective segmentation despite overlapping teeth and a low-contrast image. A small error is identified on the tip of tooth 34, where part of this segment is incorrectly classified to be a part of tooth 35. Third Row: Most teeth are segmented very accurately, although a small misalignment is present near tooth 26, where the tiny non-dental part was misclassified as tooth area. These are inconsiderate in comparison with overall model accuracy.

In the fourth row, there are more noticeable mistakes, as the radiograph is already of a more complex nature. For example, there is a slight merge of the boundary between teeth 14 and 15, reflecting some difficulty in distinguishing between closely aligned teeth. Additionally, part of tooth 12 is unsegmented, showing a potential limitation regarding handling teeth that are out of sight or with varied anatomical presentation. Despite these, the model does exceptionally well in cases of missing teeth; notice the lower arch in the fourth row, where the remaining teeth are segmented into perfection.

In general, the SE-IB-ED model presented excellent robustness against general radiographic artifacts, such as metallic restorations, overlapping teeth, noise, and poor contrast. Considering the task’s complexity, there are minor issues with respect to small errors in boundary delineation and misclassifications that occasionally happened. This suggests that generalization under such diverse and challenging conditions presents a huge opportunity for clinical applications of the model in segmenting panoramic radiographs with structural irregularities and artifacts.

## 5. Conclusions

This work presents a new method for teeth segmentation in panoramic X-ray pictures called SE-IB-ED. Extensive testing and comparison with current segmentation models show the efficiency of this new method. The SE-IB-ED model performs better than alternative designs in several measures, demonstrating its balanced sensitivity and precision in teeth segmentation tasks. Visual comparisons show how accurate the model is even when it comes close to ground truth requirements. The study’s strengths are found in its capacity to obtain good results with a small dataset, which lessens the workload associated with labeling and data gathering while preserving generalizability. Additionally, the model minimizes false positives and false negatives by maintaining a balanced ratio between accuracy and recall, and it works well when managing overlapping teeth. Nevertheless, drawbacks include the possibility of performance reduction with distorted or low-quality imagery, emphasizing how crucial it is to increase image quality. Furthermore, the training dataset’s small size probably leaves out some cases, indicating the need for larger datasets to improve the resilience of the model. Notwithstanding these drawbacks, the created system shows potential as a useful tool to support skilled dentists in activities involving the segmentation of teeth. Even though they are apt to separate and number objects on their own, some complicated situations may still require professional supervision. Subsequent research endeavors may concentrate on optimizing the model’s efficacy across diverse visual scenarios and augmenting the training dataset to enhance its applicability.

## Figures and Tables

**Table 1:** Recent work has been performed on teeth segmentation using panoramic radiographs.

Author	Dataset Used	Deep Learning Model Applied	Classification Success (Metric)
Park et al. [14]	455 panoramic radiographic images	Mask R-CNN (ResNet101 Backbone)	Tooth sampling region detection: 92.14% AP, Missing tooth region detection: 59.09% AP
Chen et al. [11]	Dental periapical films	TensorFlow, Faster R-CNN	Precision and recall >90%, IoU average value 91%
Estai et al. [12]	591 digital OPG images	U-Net, DH-R-CNN, VGG-16	Area of interest (AOI) detection 0.70 IoU, Tooth detection 0.99 recall and precision, Tooth numbering 0.98 recall, precision, and F1 score
Wu et al. [20]	Raw intraoral scans	iMeshSegNet, PointNet-Reg	iMeshSegNet: 0.964 ± 0.054 Dice similarity coefficient, PointNet-Reg: 0.597 ± 0.761 mm mean absolute error
Im et al. [15]	516 digital dental models	DG-CNN-based algorithm	Automatic tooth segmentation success rate 97.26%
Krois et al. [17]	5008 panoramic radiographic images	ResNet-34	An increase in context led to an increase in F1 score from 0.77 to 0.93
Silva et al. [21]	UFBA-UESC Dental Images	Mask R-CNN, PANet, HTC, ResNeSt	PANet: 71.3% mAP in segmentation, 74.0% mAP in numbering
Tekin et al. [23]	Bitewing dental radiographs	Mask R-CNN	Tooth detection with 100% accuracy and 97.49% mAP, Tooth numbering with 94.35% accuracy and 91.51% mAP
Chandrashekar et al. [22]	UFBA-UESC dental dataset	Faster R-CNN Mask R-CNN	Tooth segmentation 98.77%, recognition 98.44%

**Table 2:** FDI tooth numbering system.

Quadrant	Tooth Numbers
Upper Right (1)	18, 17, 16, 15, 14, 13, 12, 11
Upper Left (2)	21, 22, 23, 24, 25, 26, 27, 28
Lower Left (3)	31, 32, 33, 34, 35, 36, 37, 38
Lower Right (4)	48, 47, 46, 45, 44, 43, 42, 41

**Table 3:** Tooth segmentation performance (%) obtained using SE-IB-ED.

Tooth Number	F1-Score	mIoU	Precision	Recall	Acc
11	94.59	89.74	94.52	94.65	99.94
12	93.97	88.62	94.32	93.62	99.95
13	92.61	86.23	92.32	92.89	99.92
14	89.63	81.21	90.14	89.12	99.90
15	92.30	85.70	92.56	92.04	99.93
16	91.65	84.58	89.62	93.77	99.90
17	92.78	86.54	94.35	91.26	99.92
18	92.08	85.32	93.74	90.47	99.94
21	94.52	89.62	94.96	94.09	99.94
22	92.38	85.85	94.14	90.69	99.94
23	92.49	86.04	92.46	92.52	99.92
24	88.62	79.57	87.24	90.05	99.89
25	89.59	81.14	91.66	87.60	99.91
26	90.22	82.19	91.83	88.67	99.89
27	88.34	79.12	88.17	88.52	99.87
28	88.19	78.88	89.10	87.30	99.92
31	93.95	88.59	93.80	94.09	99.96
32	94.63	89.82	93.73	95.56	99.96
33	95.33	91.07	95.81	94.84	99.95
34	94.17	88.99	94.25	94.10	99.95
35	94.61	89.78	94.20	95.03	99.95
36	91.98	85.15	91.84	92.12	99.90
37	90.79	83.13	90.08	91.51	99.87
38	91.72	84.72	92.09	91.36	99.92
41	94.14	88.93	93.75	94.53	99.96
42	94.61	89.77	95.23	93.99	99.96
43	95.28	91.00	95.27	95.29	99.95
44	94.69	89.92	95.40	93.99	99.95
45	94.21	89.05	94.03	94.38	99.95
46	93.63	88.02	94.31	92.96	99.92
47	94.17	88.99	94.59	93.76	99.91
48	93.13	87.15	91.38	94.95	99.93
Average	92.66	86.39	92.84	92.49	99.93

**Table 4:** Comparative segmentation performance (%) obtained using Sigmoid and SoftMax activation functions for automated tooth segmentation.

Sigmoid			SoftMax		
Tooth Number	F1-Score	mIoU	Tooth Number	F1-Score	mIoU
11	94.59	89.74	11	94.42	89.42
12	93.97	88.62	12	93.70	88.14
13	92.61	86.23	13	88.61	79.55
14	89.63	81.21	14	82.71	70.52
15	92.30	85.70	15	89.60	81.16
16	91.65	84.58	16	91.28	83.96
17	92.78	86.54	17	90.58	82.79
18	92.08	85.32	18	89.71	81.35
21	94.52	89.62	21	94.61	89.77
22	92.38	85.85	22	92.71	86.41
23	92.49	86.04	23	89.90	81.66
24	88.62	79.57	24	84.99	73.90
25	89.59	81.14	25	88.28	79.02
26	90.22	82.19	26	90.98	83.45
27	88.34	79.12	27	88.96	80.11
28	88.19	78.88	28	87.00	77.00
31	93.95	88.59	31	92.77	87.28
32	94.63	89.82	32	93.92	89.34
33	95.33	91.07	33	94.99	90.46
34	94.17	88.99	34	93.03	86.97
35	94.61	89.78	35	93.65	88.07
36	91.98	85.15	36	89.46	80.93
37	90.79	83.13	37	88.67	79.64
38	91.72	84.72	38	91.18	83.79
41	94.14	88.93	41	93.31	87.46
42	94.61	89.77	42	94.20	89.05
43	95.28	91.00	43	94.66	89.86
44	94.69	89.92	44	94.07	88.81
45	94.21	89.05	45	93.94	88.58
46	93.63	88.02	46	93.31	88.24
47	94.17	88.99	47	94.52	90.42
48	93.13	87.15	48	94.45	89.48
Average	92.66	86.39	Average	91.51	84.58

**Table 5:** Confusion matrix obtained using SE-IB-ED.

Tooth Number	Background	11	12	13	14	15	16	17	18	21	22	23	24	25	26	27	28	31	32	33	34	35	36	37	38	41	42	43	44	45	46	47	48
**Background**	6,913,832.00	2551.00	2133.00	2584.00	2623.00	1747.00	3597.00	2730.00	1509.00	2301.00	1732.00	1945.00	2449.00	1575.00	3226.00	2917.00	1740.00	1693.00	2016.00	2054.00	2099.00	1572.00	2758.00	3966.00	1759.00	1737.00	1520.00	2245.00	1693.00	2021.00	2861.00	2839.00	2456.00
**11**	1388.00	36,448.00	197.00	0.00	0.00	0.00	0.00	0.00	0.00	20.00	0.00	0.00	0.00	0.00	0.00	0.00	0.00	0.00	0.00	0.00	0.00	0.00	0.00	0.00	0.00	2.00	4.00	0.00	0.00	0.00	0.00	0.00	0.00
**12**	1139.00	86.00	29,074.00	159.00	0.00	0.00	0.00	0.00	0.00	0.00	0.00	0.00	0.00	0.00	0.00	0.00	0.00	0.00	0.00	0.00	0.00	0.00	0.00	0.00	0.00	0.00	0.00	3.00	0.00	0.00	0.00	0.00	0.00
**13**	1802.00	0.00	91.00	35,299.00	1658.00	0.00	0.00	0.00	0.00	0.00	0.00	0.00	0.00	0.00	0.00	0.00	0.00	0.00	0.00	0.00	0.00	0.00	0.00	0.00	0.00	0.00	0.00	0.00	0.00	0.00	0.00	0.00	0.00
**14**	1370.00	0.00	0.00	351.00	27,929.00	1647.00	4.00	0.00	0.00	0.00	0.00	0.00	0.00	0.00	0.00	0.00	0.00	0.00	0.00	0.00	0.00	0.00	0.00	0.00	0.00	0.00	0.00	0.00	0.00	4.00	0.00	0.00	0.00
**15**	1143.00	0.00	0.00	0.00	366.00	27,775.00	543.00	0.00	0.00	0.00	0.00	0.00	0.00	0.00	0.00	0.00	0.00	0.00	0.00	0.00	0.00	0.00	0.00	0.00	0.00	0.00	0.00	0.00	0.00	1.00	0.00	0.00	0.00
**16**	1795.00	0.00	0.00	0.00	0.00	97.00	39,779.00	176.00	0.00	0.00	0.00	0.00	0.00	0.00	0.00	0.00	0.00	0.00	0.00	0.00	0.00	0.00	0.00	0.00	0.00	0.00	0.00	0.00	0.00	0.00	0.00	0.00	0.00
**17**	2031.00	0.00	0.00	0.00	0.00	0.00	1261.00	41,841.00	388.00	0.00	0.00	0.00	0.00	0.00	0.00	0.00	0.00	0.00	0.00	0.00	0.00	0.00	0.00	0.00	0.00	0.00	0.00	0.00	0.00	0.00	0.00	0.00	0.00
**18**	1847.00	0.00	0.00	0.00	0.00	0.00	0.00	224.00	23,861.00	0.00	0.00	0.00	0.00	0.00	0.00	0.00	0.00	0.00	0.00	0.00	0.00	0.00	0.00	0.00	0.00	0.00	0.00	0.00	0.00	0.00	0.00	0.00	0.00
**21**	1573.00	44.00	0.00	0.00	0.00	0.00	0.00	0.00	0.00	36,717.00	297.00	0.00	0.00	0.00	0.00	0.00	0.00	8.00	0.00	0.00	0.00	0.00	0.00	0.00	0.00	0.00	0.00	0.00	0.00	0.00	0.00	0.00	0.00
**22**	1140.00	0.00	0.00	0.00	0.00	0.00	0.00	0.00	0.00	95.00	27,763.00	1286.00	8.00	0.00	0.00	0.00	0.00	0.00	3.00	2.00	0.00	0.00	0.00	0.00	0.00	0.00	0.00	0.00	0.00	0.00	0.00	0.00	0.00
**23**	1681.00	0.00	0.00	0.00	0.00	0.00	0.00	0.00	0.00	0.00	32.00	36,138.00	2176.00	0.00	0.00	0.00	0.00	0.00	0.00	0.00	2.00	0.00	0.00	0.00	0.00	0.00	0.00	0.00	0.00	0.00	0.00	0.00	0.00
**24**	1600.00	0.00	0.00	0.00	0.00	0.00	0.00	0.00	0.00	0.00	0.00	164.00	29,274.00	1628.00	2.00	0.00	0.00	0.00	0.00	0.00	0.00	7.00	0.00	0.00	0.00	0.00	0.00	0.00	0.00	0.00	0.00	0.00	0.00
**25**	1517.00	0.00	0.00	0.00	0.00	0.00	0.00	0.00	0.00	0.00	0.00	0.00	919.00	25,902.00	380.00	0.00	0.00	0.00	0.00	0.00	0.00	0.00	0.00	0.00	0.00	0.00	0.00	0.00	0.00	0.00	0.00	0.00	0.00
**26**	2452.00	0.00	0.00	0.00	0.00	0.00	0.00	0.00	0.00	0.00	0.00	0.00	0.00	623.00	40,779.00	1441.00	0.00	0.00	0.00	0.00	0.00	0.00	0.00	2.00	0.00	0.00	0.00	0.00	0.00	0.00	0.00	0.00	0.00
**27**	2062.00	0.00	0.00	0.00	0.00	0.00	0.00	0.00	0.00	0.00	0.00	0.00	0.00	0.00	906.00	39,462.00	1410.00	0.00	0.00	0.00	0.00	0.00	0.00	3.00	0.00	0.00	0.00	0.00	0.00	0.00	0.00	0.00	0.00
**28**	1251.00	0.00	0.00	0.00	0.00	0.00	0.00	0.00	0.00	0.00	0.00	0.00	0.00	0.00	0.00	1472.00	21,569.00	0.00	0.00	0.00	0.00	0.00	0.00	0.00	19.00	0.00	0.00	0.00	0.00	0.00	0.00	0.00	0.00
**31**	860.00	0.00	0.00	0.00	0.00	0.00	0.00	0.00	0.00	0.00	0.00	0.00	0.00	0.00	0.00	0.00	0.00	20,519.00	19.00	0.00	0.00	0.00	0.00	0.00	0.00	43.00	0.00	0.00	0.00	0.00	0.00	0.00	0.00
**32**	690.00	0.00	0.00	0.00	0.00	0.00	0.00	0.00	0.00	0.00	0.00	0.00	0.00	0.00	0.00	0.00	0.00	34.00	23,705.00	28.00	0.00	0.00	0.00	0.00	0.00	0.00	0.00	0.00	0.00	0.00	0.00	0.00	0.00
**33**	1358.00	0.00	0.00	0.00	0.00	0.00	0.00	0.00	0.00	0.00	0.00	0.00	0.00	0.00	0.00	0.00	0.00	0.00	9.00	36,588.00	178.00	0.00	0.00	0.00	0.00	0.00	0.00	0.00	0.00	0.00	0.00	0.00	0.00
**34**	788.00	0.00	0.00	0.00	0.00	0.00	0.00	0.00	0.00	0.00	0.00	0.00	0.00	0.00	0.00	0.00	0.00	0.00	0.00	36.00	29,601.00	818.00	5.00	0.00	0.00	0.00	0.00	0.00	0.00	0.00	0.00	0.00	0.00
**35**	825.00	0.00	0.00	0.00	0.00	0.00	0.00	0.00	0.00	0.00	0.00	0.00	0.00	0.00	0.00	0.00	0.00	0.00	0.00	0.00	32.00	30,614.00	488.00	0.00	0.00	0.00	0.00	0.00	0.00	0.00	0.00	0.00	0.00
**36**	1772.00	0.00	0.00	0.00	0.00	0.00	0.00	0.00	0.00	0.00	0.00	0.00	0.00	0.00	0.00	0.00	0.00	0.00	0.00	0.00	0.00	85.00	44,058.00	1241.00	18.00	0.00	0.00	0.00	0.00	0.00	0.00	0.00	0.00
**37**	1447.00	0.00	0.00	0.00	0.00	0.00	0.00	0.00	0.00	0.00	0.00	0.00	0.00	0.00	0.00	0.00	0.00	0.00	0.00	0.00	0.00	0.00	1397.00	51,500.00	1494.00	0.00	0.00	0.00	0.00	0.00	0.00	0.00	0.00
**38**	1389.00	0.00	0.00	0.00	0.00	0.00	0.00	0.00	0.00	0.00	0.00	0.00	0.00	0.00	0.00	0.00	2.00	0.00	0.00	0.00	0.00	0.00	0.00	1237.00	33,372.00	0.00	0.00	0.00	0.00	0.00	0.00	0.00	0.00
**41**	785.00	0.00	0.00	0.00	0.00	0.00	0.00	0.00	0.00	0.00	0.00	0.00	0.00	0.00	0.00	0.00	0.00	21.00	0.00	0.00	0.00	0.00	0.00	0.00	0.00	20,402.00	31.00	0.00	0.00	0.00	0.00	0.00	0.00
**42**	978.00	0.00	0.00	0.00	0.00	0.00	0.00	0.00	0.00	0.00	0.00	0.00	0.00	0.00	0.00	0.00	0.00	0.00	0.00	0.00	0.00	0.00	0.00	0.00	0.00	46.00	23,930.00	50.00	0.00	0.00	0.00	0.00	0.00
**43**	1173.00	0.00	1.00	0.00	0.00	0.00	0.00	0.00	0.00	0.00	0.00	0.00	0.00	0.00	0.00	0.00	0.00	0.00	0.00	0.00	0.00	0.00	0.00	0.00	0.00	0.00	20.00	36,286.00	343.00	0.00	0.00	0.00	0.00
**44**	1377.00	0.00	0.00	0.00	0.00	0.00	0.00	0.00	0.00	0.00	0.00	0.00	0.00	0.00	0.00	0.00	0.00	0.00	0.00	0.00	0.00	0.00	0.00	0.00	0.00	0.00	0.00	62.00	31,120.00	665.00	0.00	0.00	0.00
**45**	1057.00	0.00	0.00	0.00	6.00	1.00	0.00	0.00	0.00	0.00	0.00	0.00	0.00	0.00	0.00	0.00	0.00	0.00	0.00	0.00	0.00	0.00	0.00	0.00	0.00	0.00	0.00	0.00	29.00	30,161.00	521.00	0.00	0.00
**46**	1349.00	0.00	0.00	0.00	0.00	0.00	0.00	0.00	0.00	0.00	0.00	0.00	0.00	0.00	0.00	0.00	0.00	0.00	0.00	0.00	0.00	0.00	0.00	0.00	0.00	0.00	0.00	0.00	0.00	55.00	41,272.00	1195.00	8.00
**47**	1471.00	0.00	0.00	0.00	0.00	0.00	2.00	0.00	0.00	0.00	0.00	0.00	0.00	0.00	0.00	0.00	0.00	0.00	0.00	0.00	0.00	0.00	0.00	0.00	0.00	0.00	0.00	0.00	0.00	0.00	54.00	55,358.00	1401.00
**48**	1356.00	0.00	0.00	0.00	0.00	0.00	0.00	7.00	0.00	0.00	0.00	0.00	0.00	0.00	0.00	0.00	0.00	0.00	0.00	0.00	0.00	0.00	0.00	0.00	0.00	0.00	0.00	0.00	0.00	0.00	0.00	5.00	35,524.00

**Table 6:** Comparison of performances (%) obtained using various segmentation models. SE-IB-ED vs. other architectures.

Method	F1-Score	mIoU	Precision	Recall	Acc
SE-IB-ED	92.65	86.38	92.84	92.49	99.92
Eff-U-Net	91.30	84.07	91.75	90.93	99.92
Att-U-Net	89.82	81.78	92.07	87.80	99.90
LinkNet	89.60	81.32	92.91	86.64	99.90
FPN	86.58	76.55	86.64	86.79	99.87
Trans-U-Net	74.41	60.88	75.53	79.61	99.69

**Table 7:** SE-IB-ED Model Performance Compared to Existing Dental Segmentation Models.

Author	Dataset Used	Deep Learning Model Applied	Classification Success (Metric)
Park et al. [14]	455 panoramic radiographic images	Mask R-CNN (ResNet101 Backbone)	Tooth sampling region detection: 92.14% AP, Missing tooth region detection: 59.09% AP
Chen et al. [11]	Dental periapical films	TensorFlow, Faster R-CNN	Precision and recall >90%, IoU average value 91%
Estai et al. [12]	591 digital OPG images	U-Net, DH-R-CNN, VGG-16	AOI detection 0.70 IoU, Tooth detection 0.99 recall and precision, Tooth numbering 0.98 recall, precision, and F1 score
Wu et al. [20]	Raw intraoral scans	iMeshSegNet, PointNet-Reg	iMeshSegNet: 0.964 Â ± 0.054 Dice similarity coefficient, PointNet-Reg: 0.597 Â ± 0.761 mm mean absolute error
Im et al. [15]	516 digital dental models	DG-CNN-based algorithm	Automatic tooth segmentation accuracy rate 97.26%
Krois et al. [17]	5008 panoramic radiographic images	ResNet-34	An increase in context led to an increase in F1 score from 0.77 to 0.93
Silva et al. [21]	UFBA-UESC Dental Images	Mask R-CNN, PANet, HTC, ResNeSt	PANet: 71.3% mAP in segmentation, 74.0% mAP in numbering
Tekin et al. [23]	Bitewing dental radiographs	Mask R-CNN	Tooth detection with 100% accuracy and 97.49% mAP, Tooth numbering with 94.35% accuracy and 91.51% mAP
Chandrashekar et al. [22]	UFBA-UESC dental dataset	Faster R-CNN, Mask R-CNN	Tooth segmentation accuracy 98.77%, recognition 98.44%
This Study (SE-IB-ED)	313 panoramic radiographs	SE-IB-ED (InceptionV3 Encoder)	F1-score: 92.66%, mIoU: 86.39%, Precision: 92.84%, Recall: 92.49%, Accuracy: 99.93%

## Data Availability

The raw data supporting the conclusions of this article can be made available by the authors upon reasonable request.
